# Modeling Pancreatic Cancer with Patient-Derived Organoids Integrating Cancer-Associated Fibroblasts

**DOI:** 10.3390/cancers14092077

**Published:** 2022-04-21

**Authors:** Yoon-Ha Go, Woo Hee Choi, Won Jung Bae, Sook-In Jung, Chang-Hoon Cho, Seung Ah Lee, Joon Seong Park, Ji Mi Ahn, Sung Won Kim, Kyung Jin Lee, Dakeun Lee, Jongman Yoo

**Affiliations:** 1 Department of Microbiology, CHA University School of Medicine, Seongnam 13488, Korea; dbsgk3343@gmail.com (Y.-H.G.); whchoi1204@gmail.com (W.H.C.); tnrdls2466@naver.com (S.-I.J.); 2 CHA Organoid Research Center, CHA University, Seongnam 13488, Korea; 3 R&D Institute, ORGANOIDSCIENCES Ltd., Seongnam 13488, Korea; dbsgk3343@naver.com (C.-H.C.); kjlee@organoidrx.com (K.J.L.); 4 Department of Pathology, Ajou University School of Medicine, Suwon 16499, Korea; baewj@aumc.ac.kr (W.J.B.); 500273@aumc.ac.kr (J.M.A.); 5 Department of Surgery, CHA Bundang Medical Center, CHA University, Seongnam 13496, Korea; mdseungah@chamc.co.kr; 6 Pancreatobiliary Cancer Clinic, Department of Surgery, Gangnam Severance Hospital, Yonsei University College of Medicine, Seoul 06273, Korea; jspark330@yuhs.ac; 7 Department of Otolaryngology—Head and Neck Surgery, Seoul St. Mary’s Hospital, College of Medicine, The Catholic University of Korea, Seoul 06591, Korea; kswent@catholic.ac.kr

**Keywords:** pancreatic cancer, organoid, tumor microenvironment, cancer-associated fibroblast, extracellular matrix

## Abstract

**Simple Summary:**

Pancreatic cancer tissue is resistant to anticancer drugs because of its complex microenvironment. Cancer-associated fibroblasts (CAFs) are an important source of extracellular matrix components, which alter the physical and chemical properties of pancreatic tissue, thus impairing effective intratumoral drug delivery and resulting in resistance to conventional chemotherapy. In this study, we developed a novel CAF-integrated pancreatic cancer organoid (CIPCO) model that can mimic the tumor microenvironment and confirmed that the gene expression and pathological characteristics of CIPCO are similar to those of human cancer tissue. The organoid model could serve as a preclinical model for developing individualized therapies.

**Abstract:**

Pancreatic cancer is a devastating disease and is highly resistant to anticancer drugs because of its complex microenvironment. Cancer-associated fibroblasts (CAFs) are an important source of extracellular matrix (ECM) components, which alter the physical and chemical properties of pancreatic tissue, thus impairing effective intratumoral drug delivery and resulting in resistance to conventional chemotherapy. The objective of this study was to develop a new cancer organoid model, including a fibrous tumor microenvironment (TME) using CAFs. The CAF-integrated pancreatic cancer organoid (CIPCO) model developed in this study histologically mimicked human pancreatic cancer and included ECM production by CAFs. The cancer cell–CAF interaction in the CIPCO promoted epithelial–mesenchymal transition of cancer cells, which was reversed by CAF inhibition using all-trans retinoic acid. Deposition of newly synthesized collagen I in the CIPCO disturbed the delivery of gemcitabine to cancer cells, and treatment with collagenase increased the cytotoxic effect of gemcitabine. This model may lead to the development of next-generation cancer organoid models recapitulating the fibrous TME.

## 1. Introduction

Pancreatic cancer is an aggressive tumor that is difficult to detect early and is associated with a poor prognosis [1]. Despite intense research efforts over the last several decades, many patients with pancreatic cancer do not survive >1 year after diagnosis, and the 5-year survival rate is <8% [2]. Although nucleoside analogs (e.g., gemcitabine) and combination regimens such as FOLFIRINOX are available therapies against pancreatic cancer, systemic chemotherapeutic approaches have shown unsatisfactory results and high toxicity [3]. Ex vivo cancer models that recapitulate the microenvironment (and thus pathophysiology) of pancreatic cancer are urgently needed to develop more efficient anticancer drugs against this disease.

A tumor mass consists of many types of cells, including cancer cells, endothelial cells, stem cells, cancer-associated fibroblasts (CAFs), and various immune cells, as well as extracellular matrix (ECM) components (e.g., collagen and hyaluronic acid) and secreted molecules (e.g., TIMP1 and VEGF), collectively termed the tumor microenvironment (TME) [4,5,6]. CAFs are a key component of the TME and play diverse and critical roles in cancer proliferation, immune exclusion, and resistance to anticancer medications [7,8]. CAFs are classified into three types: inflammatory (iCAFs), myofibroblastic (myoCAFs), and antigen-presenting (apCAFs) [4]. Interaction of CAFs with cancer cells affects cancer progression [9]. Therefore, CAFs are considered new potential therapeutic targets in various cancers [7,8]. Pancreatic adenocarcinoma is classified into several subtypes: basal and activated stromal types [10]; squamous, pancreatic progenitor, immunogenic, and aberrantly differentiated endocrine exocrine [11]; and four subtypes based on distinct phenotypes, including matrix- and immune-related signatures, α-smooth muscle actin expression, and proliferation rate [12]. Microdissecting cellular subtype-specific changes in gene expression provides a better understanding of the pathogenic mechanism of pancreatic cancer and may lead to the identification of novel therapeutic targets.

Diverse types of organoids have been derived directly from various human tissues [13,14]. Organoid technology has been applied to the study of cancer, and cancer organoids are attracting attention as a promising preclinical model. However, because most cancer organoids are composed of cancer cells and do not contain TME elements, cancer organoids alone do not represent the full spectrum of human cancers. Thus, disease progression or drug resistance induced by the TME cannot be properly evaluated or predicted using this simple cancer organoid model. Here, we aimed to develop a clinically relevant organoid model by integrating CAFs into pancreatic cancer organoids.

## 2. Materials and Methods

### 2.1. Three-Dimensional Organoid Culture System

This study was approved by the institutional review board (IRB) of Yonsei University Hospital (3-2017-0369) and performed after obtaining written consent from all donors. Human pancreatic tumor (adenocarcinoma) tissues were minced and washed in DMEM (Welgene, Daegu, Korea) supplemented with 1% fetal bovine serum (FBS) (GIBCO, Grand Island, NY, USA) and 1× penicillin/streptomycin (P/S) (Welgene) three times. Washed cancer tissue was digested with collagenase II (5 mg/mL, GIBCO) in DMEM/F12 (GIBCO), 10 mM HEPES (GIBCO), 1× GlutaMAX (GIBCO), and 1× P/S at 37 °C for 1 h. After digestion, isolated cells were suspended in an organoid culture medium and mixed with Matrigel (Corning, MA, USA) at a ratio of 1:1. The cells embedded in Matrigel were plated on 48-well culture plates and incubated for 20 min at 37 °C under 5% CO_2_ for polymerization of the matrices. The pancreatic organoid culture medium was composed of advanced DMEM/F12 (GIBCO) supplemented with 50% Wnt3A conditioned medium (from the L-Wnt-3A cell line, ATCC, CRL-2647), 10% R-Spondin-1 conditioned medium (from HA-R-Spondin1-Fc 293T cells, Cultrex^®^, 3710-001-01), 1× B-27 (GIBCO), 10 mM nicotinamide (Sigma-Aldrich, Chemie, Steinheim, Germany), 10 mM HEPES, 1× GlutaMAX, 1× P/S, 100 ng/mL FGF10 (NKMAX, Seongnam, Korea), 1 mM N-acetylcysteine (Sigma-Aldrich), 50 ng/mL recombinant human EGF (PeproTech, EC, London, UK), 100 ng/mL recombinant human Noggin (R&D system, Minneapolis, MN, USA), 500 nM A83-01 (Tocris Bioscience, Ellisville, MO, USA), 10 nM [Leu15]-Gastrin 1, human (Sigma-Aldrich), and 25 µg/mL Plasmocin (InvivoGen, San Diego, CA, USA). The culture medium was replaced every 2 days. Established organoids were tested for mycoplasma infection, and no infection was observed.

### 2.2. Isolation of CAFs from Pancreatic Cancer Tissue

Pancreatic cancer tissues were minced and washed with Dulbecco’s phosphate-buffered saline (DPBS) three times. Chopped tissues were cultured in DMEM supplemented with 10% FBS, 1× GlutaMAX, and 1× P/S on 12-well culture plates at 37 °C under 5% CO_2_. The phenotype of the isolated CAFs was confirmed by staining with α-smooth muscle actin and vimentin. CAFs between passages 3 and 7 were used in this study [15].

### 2.3. Co-Culture of Pancreatic Cancer Organoids (PCOs) and CAFs

For co-culture, established PCOs were roughly dissociated into clumps, and CAFs were dissociated into single cells. The dissociated PCOs (3.3 × 10^4^ cells) and CAFs (1 × 10^5^ cells) were blended at a ratio of 1:3. The cell mixture was suspended in organoid culture medium and then seeded in Matrigel at a ratio of 4:6. The final CAF-integrated PCOs (CIPCOs) were incubated in a full organoid culture medium at 37 °C under 5% CO_2_.

To confirm the effect of CAFs on increasing EMT factors, cells were pretreated with all-trans retinoic acid (ATRA) (a drug that renders CAFs ineffective [16]) for 24 h at 100 µM. The ATRA-pretreated CAFs and PCOs were co-cultured.

### 2.4. KRAS Mutation Analysis

To detect KRAS variants of human pancreatic tumor (adenocarcinoma), genomic DNA was extracted from tissues and organoids using the AccuPrep^®^ Genomic DNA Extraction Kit (Bioneer, Daejeon, Korea) according to the manufacturer’s instructions. The mutational status of KRAS was analyzed by conventional Sanger sequencing (Bioneer).

### 2.5. Histology and Immunohistochemistry

Tissue and organoids were fixed with 4% paraformaldehyde (PFA, Biosolution Co., Ltd., Seoul, Korea) for 1 day and 30 min, respectively, and embedded in paraffin. Paraffin sections (4 µm thick) were deparaffinized in xylene, hydrated in a graded series of ethanol, and then stained using H&E and a Masson’s trichrome staining kit (Dako, Carpinteria, CA, USA). For immunohistochemistry, antigen retrieval was performed by incubation in sodium citrate buffer (10 mM sodium citrate with 0.05% Tween-20, pH 6.0) at 95 °C for 1 h. Endogenous peroxidase was blocked by incubation in 3% H_2_O_2_ in methanol for 10 min. After blocking with 1% bovine serum albumin (BSA, GenDEPOT, Barker, TX, USA) in PBS, tissue sections were incubated at 4 °C overnight with a primary antibody. The primary antibodies used in this study were as follows: E-cadherin (36-B5; 1:120; Leica Biosystems, Buffalo Grove, IL, USA); N-cadherin (1:200; BD Transduction Laboratories, Franklin Lakes, NJ, USA); vimentin (V9; 1:100; Leica Biosystems); cytokeratin 19 (A53-B; 1:100; Cell Marque, Rocklin, CA, USA); Twist-1 (1:200; Invitrogen, Carlsbad, CA, USA); and hNucleoli (NM95; 1:200; Abcam, Waltham, MA, USA). Sections were then incubated at room temperature for 30 min with a biotinylated secondary antibody (1:200; anti-mouse IgG or anti-rabbit IgG) using the Vectastain ABC kit (Vector Laboratories, Burlingame, CA, USA) and stained according to the avidin-biotin complex method using the Vectastain DAB kit (Vector Laboratories).

### 2.6. Immunofluorescence

Paraffin sections were deparaffinized in xylene, hydrated in a graded series of ethanol, and blocked by incubation with 1% BSA in PBS for 2 h. The following primary antibodies were used at 4 °C overnight at the indicated dilutions: α-SMA (1:100; Biolegend); IL-6 (1:30; Invitrogen); Ki67 (SP6; 1:200; Abcam); pan-cytokeratin (AE1/AE3 + 5D3; 1:200; Abcam); E-cadherin (1:200; R&D Systems, Wiesbaden, Germany); N-cadherin (1:200; BD Transduction Laboratories, Franklin Lakes, NJ, USA); and collagen type 1 (1:200; Invitrogen). After washing with PBS, sections were incubated for 2 h at room temperature in secondary antibodies (Alexa Fluor 488 goat anti-mouse IgG, 1:400; Alexa Fluor 488 goat anti-rabbit IgG, 1:400; Alexa Fluor 488 Donkey anti-Goat IgG, 1:400; Alexa Fluor 594 goat anti-mouse IgG, 1:400; and Alexa Fluor 594 goat anti-rabbit IgG, 1:400). All secondary antibodies were purchased from Invitrogen. Phase-contrast images were acquired on a Zeiss LSM 880 inverted Confocal Laser Scanning Microscope (Carl Zeiss, Oberkochen, Germany).

### 2.7. Flow Cytometry for Cell Sorting

To confirm the changes of EMT-related genes in the CAF-integrated PCOs, the cancer cells were isolated from CIPCOs. Briefly, CIPCOs were dissociated with 0.25% trypsin-EDTA (GIBCO) and washed using DPBS. Cells were blocked at 4 °C for 30 min in 1% BSA in DPBS blocking solution and subsequently incubated with antibody against surface marker at 4 °C for 45 min. The antibody was APC-conjugated anti-CD326 (EpCAM) (1:100, Biolegend, 324208). After that, cancer cells were sorted using fluorescence-activated cell sorting (FACS) in a MoFlo-XDP high-speed cell sorter (Beckman Coulter). Single cells were collected in a 5 mL tube coated with 2% FBS in DPBS and used for quantitative reverse transcription-polymerase chain reaction (qRT-PCR) analysis.

### 2.8. Quantitative Reverse Transcription-Polymerase Chain Reaction

Total RNA was extracted from cells using the TRIzol reagent (Invitrogen) according to the manufacturer’s instructions. Then, 1 ug of RNA was used to synthesize cDNA using Accu-Power RT PreMix (Bioneer, Seoul, Korea). Quantitative PCR (qPCR) was performed with cDNA (1 µg/mL) and SYBR^®^ Premix Ex Taq™ (Takara Bio, Shiga, Japan) on a Thermal Cycler Dice Real-Time System III (Takara Bio). Relative mRNA levels of target genes were normalized and calculated with the comparative Ct method (ΔΔCt). The sequences of the specific primers used in this study are detailed in Table S1.

### 2.9. Cell Viability Analysis

Cancer cells were labeled with CellTrackerTM Green 5-chloromethyl fluorescein diacetate (CMFDA). PCOs and CIPCOs were cultured for 4 days, followed by treatment with gemcitabine (Yuhan, Seoul, Korea) with or without 100 ng/mL collagenase (type XI from *Clostridium histolyticum*, Sigma-Aldrich) for 72 h. After that, PCOs and CIPCOs were treated with propidium iodide (PI; Sigma-Aldrich), and the PI-positive area was calculated to estimate the cancer cell death in the multicellular organoid model. Fluorescence images were acquired on a Lionheart FX Automated Microscope (Biotek, Winooski, VT, USA). After 72 h of drug treatment, green and red fluorescence was detected. Z-stack images were obtained by scanning an organoid every 10 μm from the top of the organoid with a total scan range of 100 μm in depth. A plane approximately halfway through the z-stack image was chosen for quantification, and the fluorescence intensities were plotted against the radius. Area analysis was processed using Gen5 Image Prime 3.08 (Biotek).

### 2.10. In Vivo Experiments

Animal experiments were performed according to guidelines evaluated and approved by the ethics committee of CHA University (IACUC210021). For the cancer xenograft mouse model, 6-week-old nude mice (BALB/c Nude Mouse, JABIO, Korea) were used. Briefly, three organoids from each group were dissociated into clumps by TrypLE and washed with DPBS. Then, cells were injected subcutaneously into the left flank of each mouse. After 40 days, xenograft tumors were collected from mice, fixed with formalin, and analyzed by immunohistochemistry and immunofluorescence.

### 2.11. Statistical Analysis

The differences in experimental data were analyzed by two-tailed t-test or one-way ANOVA using the GraphPad Prism software package, version 5.0 (GraphPad Prism, CA, USA). All error bars indicate standard deviation unless otherwise stated. A * *p*-value < 0.05 was considered statistically significant.

## 3. Results

### 3.1. Establishment of CAF-Integrated Pancreatic Cancer Organoids

Oncogenic KRAS mutation is a major event in pancreatic cancer [17,18]. In this study, we established PCOs and isolated CAFs from human pancreatic cancer tissues, and the KRAS G12D mutation was identified in pancreatic cancer tissues and all established organoid lines (Table S2). To develop the CIPCO model, CAFs at various densities were co-cultured with PCOs, and matrix contraction mediated by the CAF-induced fibrotic stroma was observed in the CIPCO with a density of 1 × 10^5^ CAFs (Figure S1).

**Figure 1:**
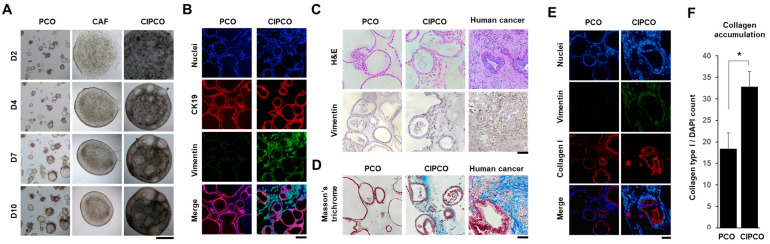
Development of cancer-associated fibroblast (CAF)-integrated pancreatic cancer organoid (CIPCO) model and its morphologic characteristics. The CIPCOs were incubated for 2–10 days at 37 °C under 5% CO_2_. CIPCO analysis was performed on day 5. (**A**) Bright-field images of pancreatic cancer organoids (PCOs), CAFs, and CIPCOs on days 2, 4, 7, and 10. Bar = 500 μm. (**B**) Immunofluorescence images of the PCO and CIPCO models labeled with CK19 and vimentin. Bar = 100 μm. (**C**) Comparison of H&E and vimentin-stained images of PCOs, CIPCOs, and human pancreatic cancer tissues. Bar = 200 μm. (**D**) Comparison of Masson’s trichrome-stained images of PCOs, CIPCOs, and human pancreatic cancer cells. Bar = 500 μm. (**E**) Immunofluorescence images of PCOs and CIPCOs labeled with collagen I. Bar = 100 μm. (**F**) Quantitative analysis of the relative fluorescence intensity of collagen I in PCOs and CIPCOs. (*n* = 6) * *p* < 0.05.

Organoids, which grow individually in PCO cultures, grew intermingled with CAFs and showed a tight organ-like spheroid structure in the CIPCO co-culture (Figure 1A). Immunofluorescence images showed cytokeratin 19 (CK19)-positive cancer glands intermixed with vimentin-positive CAFs in the CIPCO (Figure 1B) [19,20]. H&E staining detected matrices in the pink area of the CIPCO, which contained CAFs, whereas CAFs and matrix were not detected in the PCO (Figure 1C, upper images). Consistently, immunostaining for vimentin showed that CAFs were mixed with cancer glands, which mimicked the original human cancer tissues (Figure 1C, bottom images). Masson’s trichrome staining showed prominent matrices similar to those present in human pancreatic cancer only in the CIPCO but not in PCOs (Figure 1D). Whether these matrices originated from the Matrigel-induced ECM or newly synthesized ECM remained unclear. Because collagen I is a major component of the ECM in human pancreatic cancer [21] and is not present in Matrigel, we performed immunofluorescence staining for collagen I. A strong signal was detected in CIPCOs but not in PCOs (Figure 1E). Taken together, these results suggest that in the CIPCO model, cancer cells secrete ECM elements related to CAFs and contribute to the formation of a fibrotic stroma.

### 3.2. Characterization of CAFs and Cancer Cells in CIPCOs

**Figure 2:**
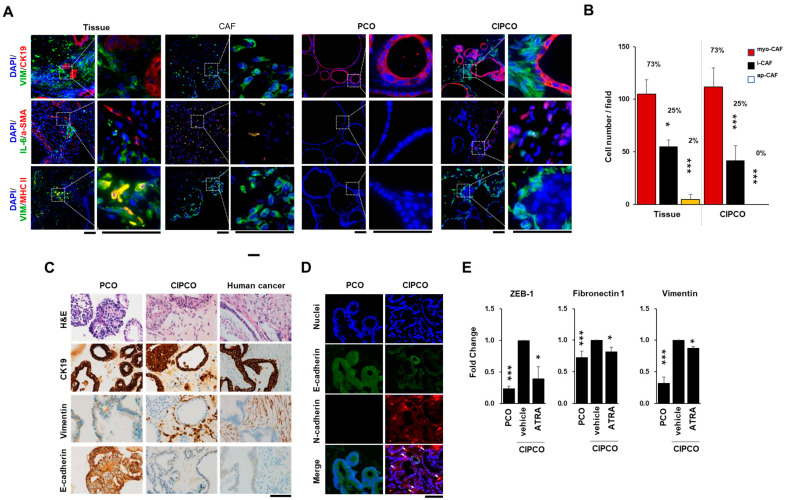
Characterization of CIPCOs. CIPCO analysis was performed on day 5. (**A**) Immunofluorescence images of human pancreatic cancer cells, CAFs, PCOs, and CIPCOs labeled with CK19, vimentin, IL-6, α-SMA, and MHC II. Bar = 100 μm. (**B**) Quantitative analysis of iCAFs and myoCAFs in PCOs, CAFs, CIPCOs, and human pancreatic cancer tissue. (*n* = 5) * *p* < 0.05, *** *p* < 0.001. (**C**) Immunohistochemical images of PCO and CIPCO models labeled with E-cadherin, CK19, and vimentin. Bar = 100 μm. (**D**) Immunofluorescence images of PCO and CIPCO models labeled with E-cadherin and N-cadherin. Bar = 100 μm. (**E**) qRT-PCR analysis of cancer cells from CIPCOs after treatment with 100 μM of all-trans retinoic acid (ATRA). (*n* = 8) * *p* < 0.05, *** *p* < 0.001.

In this study, we isolated CAFs from pancreatic cancer tissues and established a CIPCO model. To characterize the CAFs in the CIPCO, we examined the expression ratio of three CAF subtypes, myoCAF, iCAF, and apCAF. The α-SMA-positive cells and IL-6-positive cells, which identify myoCAFs and iCAFs, respectively, were detected in the CIPCO, whereas MHCII-positive cells, which indicate apCAFs, were not detected. However, MHCII was rarely expressed in tissues (Figure 2A,B). Alpha-SMA-positive cells were mainly observed in the CAFs adjacent to the organoid. The expression ratio of the three CAF subtypes was comparable to that in the original tumor (Figure 2B). IL-6-positive cells were more prevalent than α-SMA-positive cells in the CAF-only group. Cells expressing α-SMA and IL-6 were also observed in the CAF-only group but not in the CIPCO. These results suggest that the characteristics of CAFs may be changed by interactions with cancer organoids.

To characterize the cancerous properties of the CIPCO, immunohistochemical staining for CK19, vimentin, and E-cadherin was performed in PCOs, CIPCOs, and the original human cancer tissues. As shown in Figure 2C, the expression pattern of these markers in CIPCOs was similar to that in the original tumors. E-cadherin was downregulated in cancer cells in CIPCOs, consistent with its expression in original tumors. However, cancer cells in PCOs showed strong E-cadherin signals at the membrane, which is the opposite of what is seen in cancer tissue under the EMT process (Figure 2C,D). This result corresponds with previously reported studies. It has been reported that E-cadherin expression reduces in pancreatic cancers, which is one of the main characteristics of EMT [22,23]. At the same time, the EMT process provokes heterogeneous expression of E-cadherin at the tissue level [24]. Even though it is our preliminary study, we found that E-cadherin was heterogeneously expressed depending on the condition of cancer cells, even in the same glandular portions. In the original cancer tissue, cancer cells in the intraluminal papillary projections lightly expressed E-cadherin, whereas in the area surrounding the stroma did not express (data not shown). More diverse evaluations on the tissue should be performed; nevertheless, our results show that the CIPCO model recapitulates the property of human pancreatic cancer.

To confirm the changes in EMT-related genes in cancer cells induced by CAFs, PCOs were co-cultured with CAFs pretreated with ATRA. ATRA restores mechanical quiescence in pancreatic stellate cells and can be used to suppress the activity of CAFs [16]. After 5 days, FACS-sorted EpCAM-positive cancer cells from CIPCOs were analyzed by quantitative RT-PCR. Cancer cells in CIPCOs showed increased expression of ZEB-1, fibronectin, and vimentin compared with the levels in PCOs (Figure 2E). Treatment with ATRA significantly downregulated ZEB-1, fibronectin, and vimentin in cancer cells in CIPCOs. These results suggest that the EMT process in cancer cells can be attributed to CAFs. The CIPCO model was able to recapitulate the EMT process and prominent ECM, which are characteristics of human pancreatic cancer cells [6,22,25].

### 3.3. In Vivo Tumor Formation from CIPCOs

**Figure 3:**
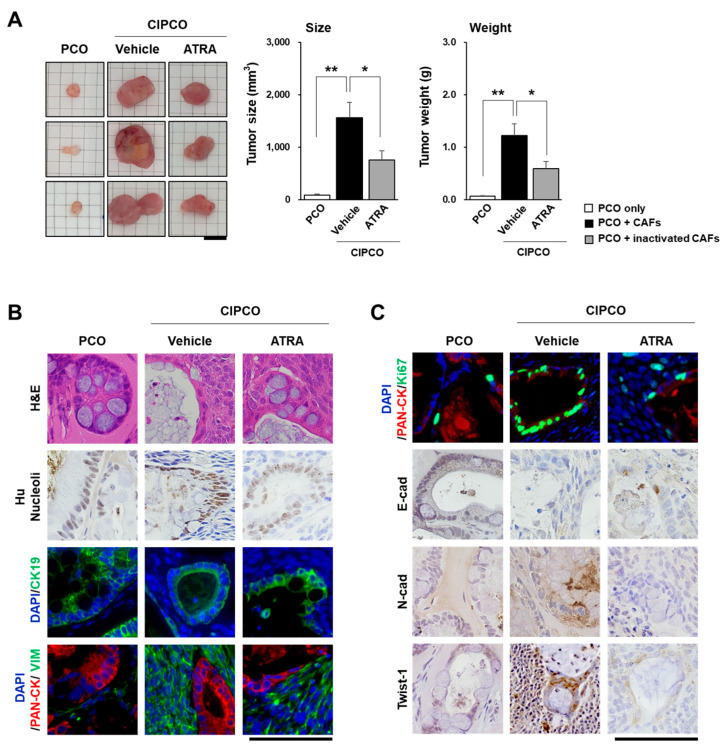
Characteristics of the tumors in xenograft models of PCOs, CIPCOs, and ATRA CIPCOs. (**A**) Comparison of tumor size and weight between the three groups. (*n* = 5) * *p* < 0.05, ** *p* < 0.01. Bar = 1 cm. (**B**,**C**) Immunofluorescence/immunohistochemical analysis of PCO, CIPCO, and ATRA CIPCO xenograft tumors. Bar = 100 μm.

To confirm the tumorigenic properties of CIPCOs in vivo, we established xenograft mouse models by subcutaneous injection of cells from PCOs and CIPCOs. At 40 days post injection, the tumor size and weight (Figure 3A) were increased in the CIPCO group compared to the other groups. Histological evaluation of xenograft tumor tissues was performed by H&E, immunofluorescence, and immunohistochemistry using cancer-specific markers. First, we used hNucleoli, CK19, and pan-cytokeratin to confirm that the tumors formed were derived from injected human pancreatic cancer cells and, using VIM, that CAFs were present in the CIPCO-injected group (Figure 3B). Then, the proliferation of CIPCO tumors could be seen more specifically using Ki67. In the CIPCO-injected group, epithelial marker E-cadherin was downregulated, and mesenchymal marker N-cadherin was upregulated. In addition, we found that there were more cells expressing Twist-1, a key transcription factor of EMT. In contrast, in the group of CIPCO with ATRA-treated CAF, the expression of Ki67 and EMT markers decreased. (Figure 3C). Collectively, these results suggest that the CIPCO model mimics the CAF-induced stroma formation and the EMT process of pancreatic cancer.

### 3.4. Differential Sensitivity to Gemcitabine between PCOs and CIPCOs

**Figure 4:**
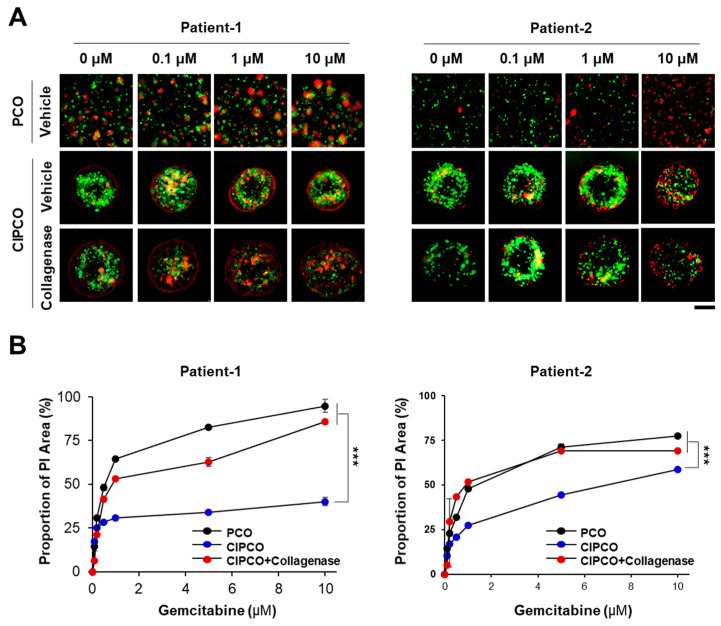
Sensitivity of cancer cells to gemcitabine in PCO and CIPCO models. (**A**) Immunofluorescence detection of propidium iodide (PI) in PCOs and CIPCOs treated with various concentrations of gemcitabine (range: 0.1–10 μM). For the CIPCO, collagenase (100 ng/mL) was added. (green: live PCOs; red: dead cells). (**B**) Quantitative analysis of PI-positive areas. PI-positive areas were calculated using Gen5 Image Prime 3.08 (Biotek). Bar = 200 μm. *** *p* < 0.001.

Drug resistance in pancreatic cancer may be caused by physical/chemical interference in the TME [9,26]. To determine whether the CIPCO model could develop drug resistance, organoids were treated for 72 h with various concentrations of gemcitabine, a therapeutic drug used in pancreatic cancer, and collagenase, which disrupts the TME. Then, PCOs and CIPCOs were stained with PI, and the PI-positive area was calculated to estimate cancer cell death. Cancer cells of PCOs were sensitive to gemcitabine, whereas cancer cells of CIPCOs were resistant to the drug (Figure 4). The addition of collagenase markedly increased the sensitivity of cancer cells to gemcitabine. This implies that the elimination of collagen from the CIPCO model increases drug uptake by cancer cells as well as drug exposure.

## 4. Discussion

The 3D tumor model is more effective for the development of medications and biomarkers than the classical 2D model using cancer cell lines [27], and organoid culture is a well-established method for modeling tumors in vitro [28,29]. Although the production of organoids from pancreatic cancer was reported previously, the inclusion of the complete architecture of the tumor microenvironment has not been reported to date [30,31,32,33,34]. In this study, we established a complex organotypic model consisting of pancreatic cancer organoids co-cultured with CAFs, which are prominent and active components of the cancer stroma.

To develop the CIPCO model, the culture conditions were optimized by controlling the density of CAFs to maximize their integration and proliferation with PCOs, resulting in the establishment of a CAF-induced fibrotic stroma (Figure S1). The use of CAFs to establish an organoid model that reflects the TME was reported previously [32,34]. Ohlund et al., identified myoCAFs, which are FAP+/α-SMA high cells located in direct proximity to neoplastic cells that form a periglandular ring surrounding cancer cell clusters. Consistent with this result obtained by co-culture of mouse pancreatic organoids and fibroblasts, we detected α-SMA expression (data not shown), indicating an activated myofibroblast-like phenotype and collagen I in CIPCOs [32]. Tsai et al., suggested a complex, patient-matched organotypic model incorporating human PCOs, CAFs, and T cells; however, the CAF-induced fibrotic stroma was not observed and failed to form TME [34].

Pancreatic cancer is characterized by a rigid fibrotic stroma that promotes cancer metastasis and drug resistance [4,12]. CAFs contribute to the formation of the fibrotic stroma by secreting cytokines that enhance cancer metastasis by inducing EMT through several pathways, including the hedgehog pathway [17,22,35]. Fibrosis increases the density of collagen, which interferes with chemokines used in T cell homing [36,37], and fibronectin deposition in the ECM accelerates the migration of tumor cells [38]. The present CIPCO model was used to demonstrate the EMT process with ECM deposition, indicating that CIPCOs can be used to study the mechanisms underlying pancreatic cancer progression by CAFs.

A dense fibrotic stroma composed of various ECM components, including collagen, laminin, and fibronectin, alters the physicochemical properties of tumors, thus impairing intratumoral drug delivery and resulting in resistance to conventional chemotherapy against pancreatic cancer [39].

A recent study showed that the stromal matrix in pancreatic cancer plays a restraining role in attenuating tumor growth [40]. This study suggested that decreasing the density of the stromal matrix may not be sufficient as an anticancer therapeutic approach, and selectively killing cancer cells after the uptake of anticancer drugs by the stromal matrix is necessary for a better outcome. This suggests that drugs need to be carefully selected to obtain a synergistic effect in pancreatic cancer.

To verify that the CIPCO model could recapitulate the alteration in drug delivery and chemoresistance induced by the ECM in human pancreatic cancer, we examined cancer cell viability in response to gemcitabine, a representative conventional chemotherapeutic agent for pancreatic cancer [41,42]. Although the induction of EMT and ECM stiffening leading to drug resistance in human pancreatic cancer tissues were reported previously [43,44,45], this is the first study to recapitulate these phenomena in an in vitro model.

## 5. Conclusions

**Figure 5:**
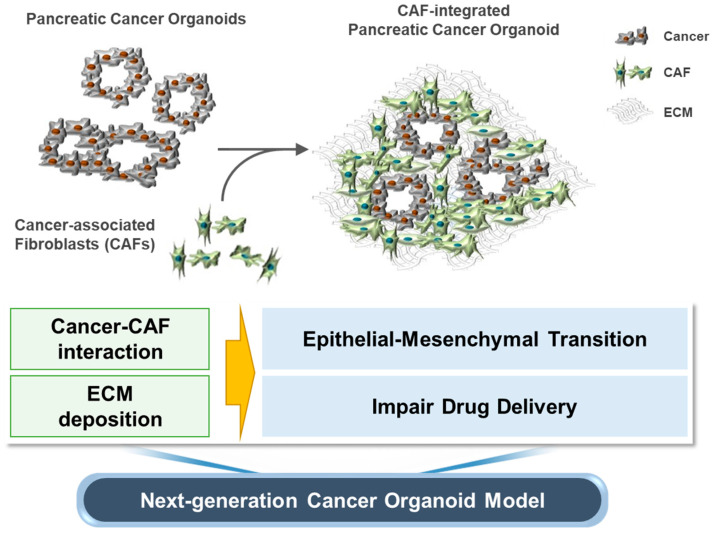
Schematic of the CIPCO model and summary of its characteristics.

In summary, we developed a new model of pancreatic cancer organoids integrated with CAFs capable of inducing a fibrous TME composed of CAFs and of producing ECM, which is not possible in conventional cancer organoid models. This novel model recapitulates various biological phenomena in pancreatic cancer, including the interaction between cancer cells and CAFs, as well as ECM-mediated interference with drug delivery (Figure 5).

The proposed model is preliminary and has two limitations. First, it does not reflect all the components of the TME, such as dendritic cells, T cells, cancer cells, B cells, macrophages, CAFs, and fibrosis. The model represents the relationship between cancer cells and CAFs. Second, the CIPCO model is not optimized for long-term expansion under co-culture conditions. We were unable to continue growing CIPCOs through passaging. However, the model provides a basis for the development of next-generation organoid models for pancreatic cancer.

## Data Availability

All data can be found in the text and supplementary.

## Supplementary Materials

The following are available online at https://www.mdpi.com/article/10.3390/cancers14092077/s1, Figure S1: Optimization of CIPCO (CAF-integrated pancreatic cancer organoid), Table S1: Primer sequences used in this study for qRT-PCR, Table S2: Clinical characteristics of two established organoid lines.

## Abbreviations

CAF	cancer-associated fibroblast
ECM	extracellular matrix
TME	tumor microenvironment
PCO	pancreatic cancer organoid
CIPCO	CAF-integrated pancreatic cancer organoid
ATRA	all-trans retinoic acid
EMT	epithelial–mesenchymal transition
PI	propidium iodide
