# Supplementary material for: Modeling Pancreatic Cancer with Patient-Derived Organoids Integrating Cancer-Associated Fibroblasts
Source: Cancers (Basel). 2022 Apr 21;14(9):2077. doi: 10.3390/cancers14092077 (PMC9103557; doi:10.3390/cancers14092077)
Supplement: Supplementary file 1 [file cancers-14-02077-s001.zip › cancers-1362772-supplementary.pdf]

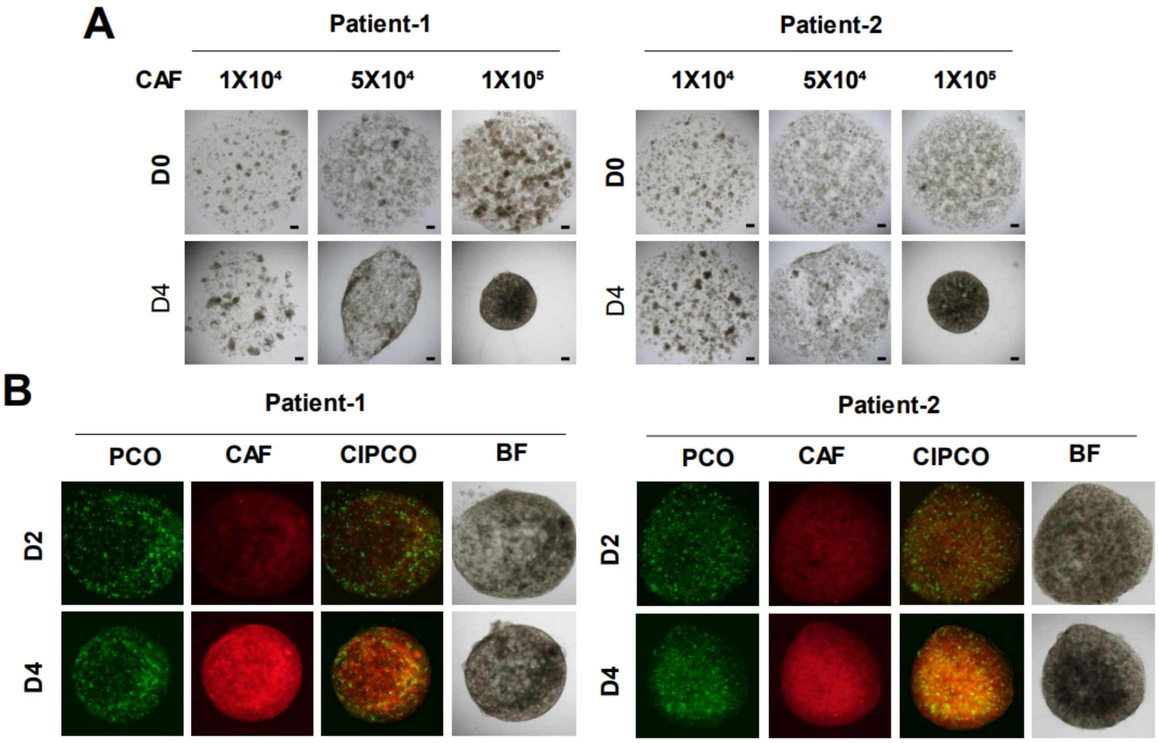

Figure S1. Optimization of CIPCO (CAF-integrated pancreatic cancer organoid).

Table S1. Primer sequences used in this study for qRT-PCR.

Gene		Sequence	Annealing temp.
ZEB-1	forward	GTGACGCAGTCTGGGTGTAA	60 °C
	reverse	CGTTTCTTGCAGTTTGGGCA	
Fibronectin	forward	CTGGGTCTCCTCCCAGAGAA	60 °C
	reverse	GGAAGGGTTACCAGTTGGGG	
Vimentin	forward	GGACCAGCTAACCAACGACA	60 °C
	reverse	AAGGTCAAGACGTGCCAGAG	

Table S2. Clinical characteristics of two established organoid lines. To detect KRAS variants, the PNAClamp™ KRAS Mutation Detection kit Ver.4 (Panagene, Inc., Daejeon, Korea) was used according to the manufacturer's protocol.

Patients	Age	Sex	Stage	KRAS mutation
P#1	58	Female	Ib	G12D (GGT → GAT)
P#2	59	Male	Ib	G12D (GGT → GAT)
P#3	73	Female	stage III	G12D (GGT->GAT)
P#4	58	Male	Iib	G12D (GGT → GAT)
P#5	82	Male	stage III	G12D (GGT->GAT)
